# Addressing ‘futility’ in psychiatry: a consensus statement

**DOI:** 10.1017/S0033291725102961

**Published:** 2026-01-14

**Authors:** Brent Michael Kious, Sarah Levitt, Sisco van Veen, Daniel Buchman, Lucy Costa, Katharina Froelich, Paul Hoff, Anna Lindblad, Suzanne Metselaar, Loïc Moureau, Gerald Neitzke, Thaddeus Mason Pope, Heidi Schenker, Julia Strupp, Manuel Trachsel, Anna Lisa Westermair

**Affiliations:** 1 Department of Psychiatry, University of Utah, Salt Lake City, USA; 2 Department of Psychiatry, University of Toronto Temerty Faculty of Medicine, Toronto, Canada; 3 Department of Psychiatry, Amsterdam University Medical Centres: Amsterdam Universitair Medische Centra, Amsterdam, Netherlands; 4 Centre for Addiction and Mental Health, Toronto, Canada; 5 Empowerment Council, Center for Addiction and Mental Health, Toronto, Canada; 6 Clinical Ethics Unit, University Hospital Basel: Universitatsspital Basel, Basel, Switzerland; 7 Swiss Academy of Medical Sciences: Schweizerische Akademie der Medizinischen Wis, Bern, Switzerland; 8 Department of Learning, Informatics, Management and Ethics, Karolinska Institute: Karolinska Institutet, Stockholm, Sweden; 9 Department of Ethics, Law, and Humanities, VU University Medical Centre Amsterdam: Amsterdam UMC Locatie VUmc, Amsterdam, Netherlands; 10 KU Leuven Faculty of Theology and Religious Studies: Katholieke Universiteit Leuven, Leuven, Belgium; 11 Institute for Ethics, History, and Philosophy of Medicine, Hannover Medical School: Medizinische Hochschule Hannover, Hannover, Germany; 12 Mitchell Hamline School of Law, St Paul, USA; 13 Praxis SULA-SUN, Solothurn, Switzerland; 14 Department of Palliative Medicine, Faculty of Medicine and University Hospital, University of Cologne: Universität zu Köln, Köln, Germany; 15 University of Basel Faculty of Medicine: Universität Basel Medizinische Fakultät, Basel, Switzerland; 16 Institute of Biomedical Ethics and History of Medicine, University of Zurich: Universität Zürich, Zürich, Switzerland

**Keywords:** ethics, futility, psychiatry, terminal mental illness, palliative care, medical aid in dying, euthanasia

## Abstract

While the concept of futility has been used widely in somatic medicine, to date, there has been limited consideration of its relevance to psychiatry. We summarize the findings of an international, multidisciplinary workshop involving clinicians, ethicists, philosophers, patient advocates, and persons with lived experience, which was focused on describing futility in psychiatry and developing ethical guidelines for making futility judgments. We outline three leading concepts of futility as they have been used in somatic medicine: physiological futility, quantitative futility, and qualitative futility. We examine the application of these concepts to the care of persons with mental illness, finding that the notion of qualitative futility is most likely to be fruitful. We consider how the concept of qualitative futility in psychiatry could relate to other ethically salient concepts such as terminal mental illness and recovery. We consider (1) who should have authority to make futility judgments in psychiatry (i.e. patients, providers, others), (2) what the process for introducing and evaluating futility judgments should be, and (3) how futility assessments should respond to patients’ goals and values. We identify potential risks of futility assessments, including psychological harms and premature treatment discontinuation, as well as potential benefits, such as reductions in harmful treatments and helpful reevaluation of the goals of care. Workshop participants regarded the concept of psychiatric futility as potentially useful. They identified how the concept could be applied to psychiatric care, as well as ethical limits on doing so.

## Introduction

There is increasing attention to how psychiatrists should care for persons with severe and persistent mental illness (SPMI) who have not benefited significantly from available treatments (Zumstein & Riese, 2020). Several factors contribute to this change. First, there is growing interest in how to care for persons with treatment-resistant psychiatric illnesses (Correll & Howes, 2021; Gaynes et al., 2020; Zhdanava et al., 2021). Second, more persons feel they are ill-served by conventional psychiatric care (Ducharme, 2023). Third, medical aid in dying (MAID) for persons with psychiatric illnesses is available in some European countries (Calati et al., 2021; Ducharme, 2023; Sheehan, Gaind, & Downar, 2017) and perhaps soon in Canada (Government of Canada, 2024). Fourth, controversy has arisen about whether severe anorexia nervosa is a terminal illness (Gaudiani, Bogetz, & Yager, 2022; Robison et al., 2024; Sharpe et al., 2023). Fifth, psychiatrists, patients, and ethicists have started considering the possibility of palliative psychiatry, an approach aimed at alleviating symptoms of SPMI that is distinct from applying psychiatry to palliative care for somatic illness (Lindblad, Helgesson, & Sjöstrand, 2019; Strand, Sjöstrand, & Lindblad, 2020; Trachsel et al., 2016a; Trachsel et al., 2016b; Westermair et al., 2022).

These five factors have led to burgeoning interest in the concept of *psychiatric futility.* In somatic medicine, an extensive literature on futility has explored the definition of futility, its scope, who has authority over it, its effects on decision-making, and whether it is a morally permissible basis for changing patient care (Bernat, 2005; Brody & Halevy, 1995; Burns & Truog, 2007; Helft, Siegler, & Lantos, 2000; Schneiderman, Jecker, & Jonsen, 1990; 1996; Tomlinson & Brody, 1990). Psychiatry is only beginning to engage in analogs of these discussions (Trachsel et al., 2019). To advance this conversation, we report the findings of an international multidisciplinary workshop regarding the nature and assessment of futility in psychiatry.

## Methods

In October, 2024, a multidisciplinary group met for a 2.5-day workshop at the Brocher Foundation in Hermance, Switzerland, to define psychiatric futility and determine how it can be identified. The eighteen international attendees included clinical ethicists, psychiatrists, patient advocates, persons with lived experience of mental illness, philosophers, health service researchers, and legal experts. Attendees were selected by the organizers (SL, MT, ALW) in view of their expertise, prior engagement with the topic, and to represent diverse perspectives and national locations.

Prior to the meeting, participants were provided with readings (Supplementary Material, Appendix 1) from a systematic literature search (conducted June 2024) and responded to the first round of a broader Delphi study exploring the concept of psychiatric futility (results pending). At the workshop, participants reviewed the results of the first Delphi round. Six participants gave brief informative talks related to futility (Table 1). The proceedings included roundtable and small group discussions about the topics outlined below. Extensive notes were recorded and provided to the first author for summary.

**Table 1.** Workshop presentations

Presentation title	Speakers
Definitions of futility in somatic medicine	Thaddeus Mason Pope
Can definitions of futility from somatic medicine be applied to mental health care?	Paul Hoff
Futility, psychiatry and patient voice	Lucy Costa
Futility in psychiatry: A concept whose time has come	Manuel Trachsel and Sarah Levitt
Is there a better alternative to the concept of futility?	Sisco van Veen
The decision-making process in forensic psychiatry for “hopeless” therapy and how to deal with it	Marc Graf

## Results

### Defining ‘futility’ in psychiatry

In somatic medicine, there are three main definitions of ‘futility’ (Table 2). All of them apply to specific treatments (Schneiderman et al., 1996). A treatment is ‘physiologically futile’ if there is *no chance* it will produce the desired physiological effect (Tomlinson & Brody, 1988). A treatment is ‘quantitatively futile’ if the likelihood of the intended effect is so low that it is not worth trying (Schneiderman et al., 1990). Finally, a treatment is ‘qualitatively futile’ if it does not seem worthwhile to the patient or provider (Brody & Halevy, 1995). These concepts were noted to be partially overlapping.

**Table 2.** Terms used

Term	Definition	Example
Physiological futility	There is *no chance* the treatment will produce the desired physiological effect	A selective serotonin reuptake inhibitor (SSRI) for the treatment of delirium tremens
Quantitative futility	The likelihood of the intended effect is so low that the treatment is not worth trying	A patient with severe obsessive–compulsive disorder did not respond to appropriate treatment with extended-release fluvoxamine; it is very unlikely that they will respond to immediate-release fluvoxamine
Qualitative futility	The expected benefits of the treatment are outweighed by its expected burdens	A patient with schizophrenia has responded partially to risperidone but feels so sedated that they cannot function; here, risperidone would be qualitatively futile
Irremediability	Global qualitative futility; the patient’s condition has not exhibited clinical response or remission despite adequate trials of all available treatments for which the expected benefit was greater than the expected burden	A patient with major depressive disorder has had years of psychotherapy of various types, adequate trials of four SSRIs, two monoamine oxidase inhibitors, two tricyclic antidepressants, augmentation with three antipsychotics, lithium, six sessions of ketamine, two separate one-month trials of transcranial magnetic stimulation, two doses of psilocybin, and 12 sessions of electroconvulsive therapy including 6 bitemporal treatments
Treatment resistance	Definition varies by condition; generally, a condition is treatment resistant if it has failed to remit or respond adequately to a sequence of treatments that would be expected to produce significant clinical benefit in most patients	A patient with major depressive disorder who did not benefit from to two adequate trials of SSRIs
Personal recovery	A process of change through which individuals improve their health and wellness, live a self-directed life, and strive to reach their full potential^35^	A person with bipolar disorder values staying employed and has managed to keep and maintain a job for several years while managing their symptoms enough that they do not get in the way of work
Lack of clinical indication	Inappropriate treatment; a treatment is not clinically indicated or inappropriate if the expected benefits of treatment, given the patient’s likely diagnosis, are outweighed by the expected burdens	The use of mixed amphetamine salts to treat negative symptoms of schizophrenia
Palliative psychiatry	An approach to psychiatric care that emphasizes reducing suffering and improving quality of life rather than promoting clinical remission or response	An intensive outpatient team discontinues an intramuscular antipsychotic injection as the patient hated its administration and it led to conflict between them and the team. Now, without the injection acting as a point of contention, the patient is willing to participate in social programming with team members, which has improved their QOL

The workshop considered whether these accounts of futility apply in psychiatry. Some workshop participants thought a psychiatric treatment can be physiologically futile if there is good evidence that it is not mechanistically appropriate. For instance, there are psychopharmacological reasons to think stimulants will not improve auditory hallucinations in schizophrenia, making them physiologically futile for this purpose (Schneiderman et al., 1990; Schneiderman & Jecker, 1995). A difficulty in applying physiological futility in psychiatry is that neuroscientific explanations of psychiatric illnesses are often lacking. Some participants thought that physiologic futility is inapplicable to psychiatry because there is always some chance a patient’s condition will respond to a treatment, even if only because of nonspecific effects.

Participants regarded physiological futility as a subtype of quantitative futility. Quantitative futility is more applicable to psychiatry, since many psychiatric treatments have such a low chance of achieving the intended effect, in a specific case, that they are not worth trying. For instance, it is unlikely that immediate-release fluvoxamine would help a patient with obsessive–compulsive disorder who showed no response to extended-release fluvoxamine. Similarly, cognitive–behavioral therapy is unlikely to help a patient who showed no response to the same protocol only a month earlier. A problem with quantitative futility, however, is that the threshold for how unlikely the intended effect of a treatment must be to make it not worth trying is debatable and value-laden.

Participants agreed that a treatment should be judged qualitatively futile if and only if its expected benefits are less than its expected burdens (i.e. adverse effects, inconvenience to the patient, expense). Expected benefits and expected burdens can be calculated as the probability of each outcome multiplied by its respective value. Participants regarded qualitative futility as applicable to psychiatry and noted that it avoids shortcomings of physiological and quantitative futility while encompassing those concepts. Qualitative futility automatically provides a threshold at which the probability of an intended effect is so low that it is not worth trying: when expected burdens outweigh expected benefits. Workshop participants unanimously agreed that just because a treatment is futile now does not guarantee it will be futile in the future. The benefit/burden tradeoff may rebalance with changes in treatment goals, the patient’s responsiveness to treatment and side effects, and the costs or accessibility of treatment.

Most workshop participants thought that qualitative futility should primarily reflect a patient’s goals. In many cases, treatment is futile because, while it promotes some goal, it is not the patient’s goal. For instance, suppose a patient prioritizes being alert over reducing hallucinations. Antipsychotics which reduce hallucinations but also produce severe sedation could be qualitatively futile for them. Conversely, if a treatment is typically futile, but a particular patient’s values mean that the benefits are greater than the burdens for them, then the treatment is not futile for them. This is not to suggest that a patient’s desire to have some treatment that is very unlikely to promote their goal (e.g. a prescription for fluvoxamine to use if they develop COVID-19) entails that the treatment is not futile for them. A patient’s preferences only make a treatment non-futile if the treatment is likely to promote what they see as good for them on balance.

In contrast, however, some workshop participants argued that a patient’s treatment goals should not contribute to futility judgments if they are unreasonable or inappropriate. Goals can be unreasonable or inappropriate if they are (1) extremely unrealistic, (2) based on factual misunderstanding, (3) not in keeping with the aims of medicine, or (4) unethical. Consider a patient with schizophrenia who does not wish to take antipsychotics only because they strongly desire not to enrich pharmaceutical companies, as they believe those companies have conspired to implant electrical devices in their body. Since the patient’s goal is manifestly unreasonable, if the benefits of antipsychotics otherwise outweigh the burdens, the treatment would not be futile. Similarly, if a patient with depression would benefit from psychotherapy, but refused psychotherapy arbitrarily (for no good reason), one might hesitate to say that psychotherapy was futile for them. Simultaneously, if treatment only serves to promote a patient’s goal, but that goal seems clinically inappropriate, the treatment would still be futile. For example, if a patient wishes to take testosterone merely to increase muscle mass, but this is likely to cause severe emotional dysregulation and liver toxicity, the psychiatrist may regard the treatment as qualitatively futile (though this may instead be regarded as ‘inappropriate’ – see below).

Several workshop participants raised concerns about this approach, however. They observed that it is controversial whether a goal is reasonable or not. Often, a patient’s goal may seem unreasonable simply because it is uncommon or unpopular. They noted that the goals of medicine are also controversial. These objections may imply that futility judgments should reflect only the patient’s goals as they stand, irrespective of whether they seem ‘reasonable’. Alternatively, they may suggest that futility is irreducibly perspectival, so that the physician, patient, and others merely have different ideas based on their individual assessments.

Another contentious question for the workshop was whether a beneficial treatment could be considered qualitatively futile if it would be unaffordable for the healthcare system or would require reallocation of scarce resources from other patients. Many workshop participants regarded broader social considerations as irrelevant to medical decisions that ought to be individualized. But some felt that if any values other than the patient’s values (like the goals of medicine) are relevant to qualitative futility, then other widely accepted moral and social values should also be considered, so that cost and scarcity could make a treatment futile.

All the definitions of futility mentioned so far apply to specific treatments rather than global assessments of a patient’s condition. Some workshop participants regarded this as a limitation, since to them ‘futility’ implies a sense of despair about the patient’s condition. Global judgments matter because they can prompt a reevaluation of the goals of care. Some participants suggested that global futility judgments – in effect, judgments that the patient’s condition is irremediable (van Veen et al., 2022) – are merited if all available medical treatments aimed at remission or symptomatic improvement are individually qualitatively futile. Other participants worried that such composite judgments still do not capture the severity of suffering and sense of hopelessness that exist when we describe a patient’s overall predicament as ‘futile’. Some workshop participants also suggested that global futility judgments would usually be inappropriate, since there is invariably *something* that could be done to promote the patient’s goals or to improve their condition. Even these participants, however, seemed to accept that for some persons, it is possible for an illness to be irremediable in the sense that improvements in its core symptoms are very unlikely.

Workshop participants considered how treatment nonadherence and refusal affect futility. If a treatment would have improved a patient’s condition if used consistently, but it was refused or used inconsistently, would it be futile if used again? This question was acknowledged to be complicated by emerging neurobiological evidence about the contribution of a ‘kindling effect’ or the impacts of a ‘prolonged duration of untreated psychosis’ to the emergence of treatment resistance (Post, 2007; Cotter et al., 2017). More crucially, can lack of adherence contribute to a judgment that the patient’s condition is irremediable? These questions are particularly relevant to psychiatric futility in Canada, where patients may ultimately be able to request MAID despite refusing treatments they regard as unacceptable (Canada, 2022). The answers may, again, depend on the reasons for nonadherence or refusal. If a patient did not adhere because they were forgetful, but memory aids would increase adherence, future treatment would not be futile. In contrast, if a patient refused treatment because they reasonably saw it as not beneficial, it may still be futile. The quality of the patient’s reasons may matter. Some participants thought that if the patient refused the treatment because of an unreasonable weighing of its burdens and benefits, a futility designation would be inappropriate.

### Relationship to other concepts

To evaluate whether the concept of psychiatric futility could be useful, workshop participants examined its relationship to similar concepts, including clinical indication, recovery, treatment resistance, and terminal mental illness (Levitt & Buchman, 2021).

Some participants felt that a treatment-specific concept of futility added little to the concept of a lack of clinical indication or ‘inappropriate’ treatment, since a treatment is not indicated if its benefits and burdens do not justify providing it – in which case it is also qualitatively futile. But futility and lack of indication may differ in several respects. First, clinical indication may be constrained by institutional recommendations, such as Food and Drug Administration (FDA) or European Medicines Agency (EMA) product information (EMA, 2025; FDA, 2024). Clinical indication may also consider the standard of care, which reflects typical physician practice in a community (Panagiotou, 2018). In that case, a treatment could be ‘not indicated’ and not futile, or futile but still ‘indicated’, since technical indication and the standard of care may not track individual benefit. For instance, yet another selective serotonin reuptake inhibitor may be technically indicated for a patient whose depression has not responded to four such medications, even though it would probably be futile (Thase & Rush, 1997).

Another concept related to futility is personal recovery (Rossi et al., 2018; Schneiderman & Jecker, 1995). ‘Recovery’ is often defined as ‘a process of change through which individuals improve their health and wellness, live a self-directed life, and strive to reach their full potential’ (Howes, Thase, & Pillinger, 2022). This definition emphasizes that recovery should reflect an individual’s goals. Thus, and somewhat surprisingly, the concept of qualitative futility coheres with recovery-oriented care, since it considers and supports the patient’s goals and values. Recognizing qualitative futility may even promote recovery in this sense, since it can shift attention to other treatments or prompt the patient to reevaluate some goals.

The concept of treatment resistance is clearly related to futility. While it has multiple definitions that vary by psychiatric condition, generally, a person’s illness is considered treatment-resistant if they have not shown symptomatic response or remission despite receiving sequential treatments that would produce the desired outcome in most patients (Howes et al., 2022). When a person has a treatment-resistant condition, the chance that future treatments are futile may be increased, though this is not guaranteed. Simultaneously, a particular treatment may be futile for a particular patient without treatment resistance if it would be inconsistent with their goals.

In somatic medicine, futility is connected to the concept of terminal illness. In psychiatry, Gaudiani et al. (2022) proposed that anorexia nervosa can be terminal, if a variety of conditions are met, including that there is ‘consistent, clear expression … that they understand further treatment to be futile, they choose to stop trying to prolong their lives, and they accept that death will be the natural outcome’ (Gaudiani et al., 2022). Clearly, Gaudiani et al. (2022) conceive of terminal anorexia as involving futility but do not define what ‘futile’ means. While many workshop participants rejected the notion of terminal psychiatric illness, the concept of futility used by Gaudiani et al. (2022) is probably like irremediability. The idea that the patient ‘understand(s) further treatment to be futile’ may suggest that given their goals, the burdens of available treatments outweigh their benefits. What Gaudiani et al. (2022) call ‘terminal anorexia’ could be less misleadingly labeled ‘irremediable anorexia nervosa’.

### Processes, risks, and benefits of futility judgments

Most workshop participants thought that if futility judgments are to be used in psychiatry, we need well-developed processes not only to ensure their accuracy and impartiality but also to minimize their risks. There are many such risks. Most obviously, futility judgments can be incorrect, leading to potentially helpful treatments being dismissed. Psychiatric prognosis is difficult (Fusar-Poli, Hijazi, Stahl, & Steyerberg, 2018; Meehan et al., 2022). It can be hard to predict when an individual with treatment-resistant illness will finally respond, recover spontaneously, or improve because of environmental changes. This is especially true for irremediability judgments, which require determining whether all available treatments are futile.

Most workshop participants emphasized that both treatment-specific futility judgments and irremediability judgments do not require certainty – only a reasonable degree of confidence that the burdens of treatments outweigh their benefits. In this respect, futility judgments are no different than other judgments in medicine, where treatment decisions are typically based on probabilities and values (Gillett, 2004).

Futility judgments could have negative psychological consequences for patients. Even if the psychiatrist merely suggests that a specific treatment is futile, the patient may hear that no treatment will help. Or the patient may take the psychiatrist to be saying that the patient themself is valueless or undeserving. This concern is even more present in global irremediability judgments, given how patients’ identities can become entangled with their illnesses (O’Connor, Kadianaki, Maunder, & McNicholas, 2018).

To minimize unintended psychological harm, workshop participants recommended (1) that treatment-specific futility judgments should be carefully prefaced and explained, (2) that the availability of other treatments should be emphasized, and (3) that the psychiatrist should repeatedly affirm their commitment to the patient as a valuable individual whose goals matter. Due to their greater epistemic threshold and potentially greater impact, global irremediability judgments require additional precautions. The psychiatrist, patient, and other members of the treatment team should carefully review prior treatments and remaining treatment options to determine the adequacy of treatment and reasons for insufficient benefit. They should also seek consultation with other experts, including patient advocates, other psychiatrists, and ethicists.

Some participants thought that when a patient’s symptoms prove to be extraordinarily treatment resistant, the psychiatrist could, and even should, cautiously raise the possibility of irremediability to minimize ongoing harm. But most workshop participants disagreed, holding that psychiatrists should not unilaterally introduce irremediability judgments, since doing so would undermine the patient’s authority about their goals and could sap the motivation of an otherwise engaged patient. This leaves some uncertainty about what providers may do when a patient is unable to articulate personal goals, but in such cases, perhaps, providers could take more initiative. Workshop participants mostly agreed that even when the patient introduces the question of irremediability or broaches a general sense of hopelessness about treatment, the psychiatrist should approach the issue carefully, as the patient may be testing the psychiatrist’s commitment to the relationship or indirectly seeking reassurance. Then, too-ready agreement with the patient’s judgment could have disastrous consequences.

At the same time, utility judgments can have benefits. They require close collaboration between patients, their supporters, and providers, potentially fostering closer therapeutic relationships. A futility judgment itself could reaffirm and strengthen the provider–patient relationship by emphasizing the provider’s commitment to the patient’s well-being. If the possibility of psychiatric futility were more broadly recognized, it could also prompt patients and providers to talk about treatment challenges and closely examine iatrogenic harm. Psychiatrists sometimes feel compelled to offer treatments that they recognize are very unlikely to be helpful (Dorfman et al., 2024). Attention to treatment-specific futility could help patients avoid unhelpful and overly burdensome treatments, resisting psychiatry’s tendency toward polypharmacy and acknowledging patients’ experiences of iatrogenic harm (Sarkar, 2017).

In cases where irremediability is suspected and clinical response and remission seem unattainable without excess treatment burden, other goals such as improved psychosocial functioning or reduced suffering may still be worth pursuing. Persons with irremediable conditions should still receive care intended to relieve their suffering, even if it does not treat the underlying condition (Moonen, Lemiengre, & Gastmans, 2016; Yager, 2021). Finally, acknowledging irremediability could provide much-needed validation for patients and psychiatrists beset by doubts about the value of ongoing treatment.

### Recommendations for future research

Our workshop identified several lacunae in our understanding of psychiatric futility, which point to five key areas for future research.

First, consideration of psychiatric futility highlights the importance of clinical research on treatment-resistant conditions. Before making any futility judgment, psychiatrists should review and implement protocols for the care of persons with treatment-resistant illness. Unfortunately, many of these protocols are underdeveloped and provide scant guidance when a patient’s condition is not merely treatment-resistant but extremely so. Consider major depression: While depression is usually called ‘treatment resistant’ after a person has not responded to merely two treatments in a single depressive episode (Sarkar, 2017), and while there are many other possible treatments for treatment-resistant depression, we know very little about which subsequent treatments we should recommend and in what order.

A second key area for research about psychiatric futility is the evaluation of the concept by representative groups of key interest holders. It is conceivable that futility judgments increase stigma or are otherwise unwelcome to patients. Qualitative research involving persons with lived experience of mental illness should explore their reactions to the concept, their ideas about when futility exists for persons with different psychiatric conditions, about best practices for futility determinations, and about future care directions for persons who have confronted futility. Similarly, research should continue to evaluate how psychiatrists think about futility and related concepts (Dorfman et al., 2024; Stoll et al., 2022; Trachsel et al., 2019).

Third, if the concept of futility gains traction in psychiatric settings and starts to influence patients’ and physicians’ decisions, it will also be important to evaluate its effects and to determine best practices for its assessment. How often do patients continue to receive treatments even though those treatments are deemed futile? What are the best practices in reevaluating treatment-specific futility judgments in the context of changing circumstances? How often are these judgments a point of disagreement in the therapeutic relationship, and how often are they a matter of consensus?

Fourth, numerous questions also arise with respect to irremediability. When shared decision-making determines that a person’s condition is irremediable, what happens to that person? How does this assessment affect the relationships between patients, their support persons, and their care teams? What is the effect of the assessment on their morbidity, mortality, and quality of life?

Fifth, and in tandem with answering the questions above, psychiatry must delineate guidance for how to respond to persons determined to have irremediable conditions. In somatic medicine, if a patient’s condition is irremediable, this often means a change in the goals of care from cure and remission to palliation. While some argue that the difference between palliative psychiatry and general psychiatry is unclear (Kious & Nelson, 2023), a shift toward a palliative mindset, occasioned by a designation of irremediability, may nevertheless open new and different approaches to care (Westermair et al., 2022). What such approaches should include is something for patients, psychiatrists, and others to determine.

## Discussion

Here, we have reported the findings of a multidisciplinary group of physicians, clinical ethicists, persons with lived experience of mental illness, patient advocates, philosophers, and legal scholars, regarding potential definitions and applications of the idea of futility in psychiatry. The aim of this report was to describe conceptual issues and identify open questions related to evaluating futility, thereby laying the foundations for future practice, research, and policy.

Strengths of these findings and recommendations include that they were developed through consensus-oriented, cooperative discussion by a group of individuals who have demonstrated expertise in the topic. Obvious limitations attend this approach. First, despite agreement in many domains, uncertainty and disagreement persist. Second, our findings may not capture the diversity of views that may exist among patients, their support persons, ethicists, psychiatrists, and other interest holders. Finally, all attendees were affiliated with Western healthcare systems in high-income countries, so we do not expect to capture views represented in non-Western contexts or in low- and middle-income countries.

## Conclusions

Some persons with severe and extremely treatment-resistant psychiatric illness will recover or even achieve symptomatic remission. Others risk receiving treatment that is unhelpful and harmful, leading them, their support persons, and their psychiatrists to doubt the value of ongoing treatment or to feel antagonistic toward the mental health system generally. The intent of the workshop was to better articulate the notion that psychiatry lacks a concept delineating its own limits and acknowledging its risk of iatrogenic harm. ‘Futility’ might offer a way of expressing these ideas. This report represents a beginning for the exploration of futility in psychiatry and raises many questions, highlighting the need for further normative and empirical research.

## Supporting information

10.1017/S0033291725102961.sm001 Kious et al. supplementary material Kious et al. supplementary material

## Supplementary material

The supplementary material for this article can be found at http://doi.org/10.1017/S0033291725102961.
