# Supplementary material for: Addressing ‘futility’ in psychiatry: a consensus statement
Source: Psychol Med. 2026 Jan 14;56:e16. doi: 10.1017/S0033291725102961 (PMC12885334; doi:10.1017/S0033291725102961)
Supplement: Kious et al. supplementary material [file S0033291725102961sup001.docx]

**Appendix 1: Recommended Readings for Workshop Participants**

Aftab, A. (2023). What should clinicians know about palliative psychopharmacology?. *AMA Journal of Ethics*, *25*(9), E710–E717. https://doi.org/10.1001/amajethics.2023.710

Austelle, C. W., Ehrie, J., & Zabinski, J. S. (2024). Hope in the face of "futility": considering the full scope of psychiatric treatment options. *AJOB Neuroscience*, *15*(1), 59–61. https://doi.org/10.1080/21507740.2023.2292496

Ben-Dor, G. A., Alebdy, D., & Xu, Y. E. (2024). Psychiatric care when cure is no longer the goal: A call for expansion of management options for treatment-resistant mental illness. *AJOB Neuroscience*, *15*(1), 70–72. https://doi.org/10.1080/21507740.2023.2292494

Brodwin, P. (2011). Futility in the practice of community psychiatry. *Medical Anthropology Quarterly*, *25*(2), 189–208. https://doi.org/10.1111/j.1548-1387.2011.01149.x

Bruni, T., & Weijer, C. (2015). A misunderstanding concerning futility. *American Journal of Bioethics*, *15*(7), 59–60. https://doi.org/10.1080/15265161.2015.1039728

Cholbi, M. J. (2013). The terminal, the futile, and the psychiatrically disordered. *International Journal of Law and Psychiatry*, *36*(5-6), 498–505. https://doi.org/10.1016/j.ijlp.2013.06.011

Cooper, R. B., Levitt, S. E., & Buchman, D. Z. (2024). Humanizing patients and their needs might affect psychiatrists' thinking about futility. *AJOB Neuroscience*, *15*(1), 64–67. https://doi.org/10.1080/21507740.2023.2292510

Coulter, A., Schuermeyer, I., & Sola, C. (2021). Evaluating ineffective treatments: A proposed model for discussing futility in psychiatric illness. *Harvard Review of Psychiatry*, *29*(3), 240–245. https://doi.org/10.1097/HRP.0000000000000293

Crow, S. J. (2023). Terminal anorexia nervosa cannot currently be identified. *International Journal of Eating Disorders*, *56*(7), 1329–1334. https://doi.org/10.1002/eat.23957

Dembo, J. S. (2010). Addressing treatment futility and assisted suicide in psychiatry. *Journal of Ethics Mental Health, 5*(1), 1-3.

Dorfman, N. J., Blumenthal-Barby, J., Ubel, P. A., Moore, B., Nelson, R., & Kious, B. M. (2024). What do psychiatrists think about caring for patients who have extremely treatment-refractory illness?. *AJOB Neuroscience*, *15*(1), 51–58. https://doi.org/10.1080/21507740.2023.2225467

Elwyn, R. (2023). A lived experience response to the proposed diagnosis of terminal anorexia nervosa: Learning from iatrogenic harm, ambivalence and enduring hope. *Journal of Eating Disorders*, *11*(1), 2. https://doi.org/10.1186/s40337-022-00729-0

Elwyn, R., Adams, M., Sharpe, S. L., Silverstein, S., LaMarre, A., Downs, J., & Burnette, C. B. (2024). Discordant conceptualisations of eating disorder recovery and their influence on the construct of terminality. *Journal of Eating Disorders*, *12*(1), 70. https://doi.org/10.1186/s40337-024-01016-w

Forbes, D. A. (2020). Futility in adolescent anorexia nervosa and the question of withdrawal of care. *Journal of Paediatrics and Child Health*, *56*(1), 5–7. https://doi.org/10.1111/jpc.14659

Gaudiani, J. L., Bogetz, A., & Yager, J. (2022). Terminal anorexia nervosa: Three cases and proposed clinical characteristics. *Journal of Eating Disorders*, *10*(1), 23. https://doi.org/10.1186/s40337-022-00548-3

Geppert, C. M. (2015). Futility in chronic anorexia nervosa: A concept whose time has not yet come. *The American Journal of Bioethics: AJOB*, *15*(7), 34–43. https://doi.org/10.1080/15265161.2015.1039720

Geppert, C. (2021). Psychotherapeutic futility. In M. Trachsel, J. Gaab, N. Biller-Andorno (Eds.) & Ş. Tekin & J. Z. Sadler (Ed.), *The Oxford Handbook Of Psychotherapy Ethics* (pp. 447–460). Oxford, United Kingdom: Oxford University Press.

Giordano, S., Goss, K. P., & Fox, J. R. E. (2012). Treating eating disorders: Some legal and ethical issues. In *Eating and Its Disorders* (pp. 102–116). Chichester, United Kingdom: John Wiley & Sons, Ltd. <https://doi.org/10.1002/9781118328910.ch7>

Giordano, S. (2019). Anorexia nervosa: a case for exceptionalism in ethical decision making. *Philosophy, Psychiatry, & Psychology*, *26*(4), 315-331.

Goldberg, J. F. (2018). When further pharmacotherapy seems futile. *Journal of Clinical Psychiatry*, *80*(1), 22421.

Kirby, J. (2021). Reconceptualizing 'psychiatric futility': could harm reduction, palliative psychiatry and assisted dying constitute a three-component spectrum of appropriate practices?. *American Journal of Bioethics*, *21*(7), 65–67. https://doi.org/10.1080/15265161.2021.1926588

Levitt, S., & Buchman, D. Z. (2021). Applying futility in psychiatry: A concept whose time has come. *Journal of Medical Ethics*, *47*(12), E60-E60. https://doi.org/10.1136/medethics-2020-106654

Lopez, A., Yager, J., & Feinstein, R. E. (2010). Medical futility and psychiatry: Palliative care and hospice care as a last resort in the treatment of refractory anorexia nervosa. *International journal of Eating Disorders*, *43*(4), 372–377. https://doi.org/10.1002/eat.20701

Mackenzie, R. (2015). Ms X: A promising new view of anorexia nervosa, futility, and end-of-life decisions in a very recent English case. *American Journal of Bioethics,* *15*(7), 57–58. https://doi.org/10.1080/15265161.2015.1042992

McKinney, C. (2015). Is Resistance (N)ever Futile? A Response to "Futility in chronic anorexia nervosa: A concept whose time has not yet come" by Cynthia Geppert. *American Journal of Bioethics*, *15*(7), 53–54. https://doi.org/10.1080/15265161.2015.1042991

Moseley, D. D. (2024). What is futility in psychiatry? *AJOB Neuroscienc*e*,* 15(1), 67–69. doi:10.1080/21507740.2023.2292508

Moureau, L., Verhofstadt, M., & Liégeois, A. (2023). Mapping the ethical aspects in end-of-life care for persons with a severe and persistent mental illness: A scoping review of the literature. *Frontiers in Psychiatry*, *14*, 1094038. https://doi.org/10.3389/fpsyt.2023.1094038

Muscatello, M. R. A., Zoccali, R. A., & Bruno, A. (2020). Is there a time when prescribing pharmacotherapy in psychiatry is futile?. *Expert Opinion on Pharmacotherapy*, *21*(7), 733–735. https://doi.org/10.1080/14656566.2020.1729739

Papathanasiou, C., & Stylianidis, S. (2022). Experiences of futility among nurses providing care to patients with borderline personality disorder in the greek mental health system. *Journal of Psychosocial Nursing and Mental Health Services*, *60*(6), 33–42. https://doi.org/10.3928/02793695-20211119-02

Paris, J. J., & Hawkins, A. (2015). "Futility" Is a failed concept in medical decision making: Its use should be abandoned. *American Journal of Bioethics*, *15*(7), 50–52. https://doi.org/10.1080/15265161.2015.1039735

Pienaar, W. (2016). Developing the language of futility in psychiatry with care. *South African Journal of Psychiatry*, *22*(1), 978. https://doi.org/10.4102/sajpsychiatry.v22i1.978

Pies, R. W. (2015). Anorexia nervosa, "futility," and category errors. *American Journal of Bioethics*, *15*(7), 44–46. https://doi.org/10.1080/15265161.2015.1039734

Pozón, S. R. (2022). Futility and palliative psychiatry in mental health: New clinical and ethical challenges. *Revista Colombiana de Psiquiatria*, *51*(2), 87–88. https://doi.org/10.1016/j.rcpeng.2022.06.001

Rosenbaum, D. M. S., Robertson, D., & Law, S. (2022). Psychiatric futility and palliative care for a patient with clozapine-resistant schizophrenia. *Journal of Psychiatric Practice*, *28*(4), 344–348. https://doi.org/10.1097/PRA.0000000000000645

Trachsel, M., Hodel, M. A., Irwin, S. A., Hoff, P., Biller-Andorno, N., & Riese, F. (2019). Acceptability of palliative care approaches for patients with severe and persistent mental illness: a survey of psychiatrists in Switzerland. *BMC Psychiatry*, *19*(1), 111. https://doi.org/10.1186/s12888-019-2091-x

Trachsel, M., Irwin, S. A., Biller-Andorno, N., Hoff, P., & Riese, F. (2016). Palliative psychiatry for severe persistent mental illness as a new approach to psychiatry? Definition, scope, benefits, and risks. *BMC Psychiatry*, *16*, 260. https://doi.org/10.1186/s12888-016-0970-y

Trachsel, M., & Jox, R. J. (2022). Suffering is not enough: Assisted dying for people with mental illness. *Bioethics*, *36*(5), 519–524. https://doi.org/10.1111/bioe.13002

Trachsel, M., Wild, V., Biller-Andorno, N., & Krones, T. (2015). Compulsory treatment in chronic anorexia nervosa by all means? Searching for a middle ground between a curative and a palliative approach. *American Journal of Bioethics*, *15*(7), 55–56. https://doi.org/10.1080/15265161.2015.1039730

Westermair, A. L., & Trachsel, M. (2023). Moral intuitions about futility as prompts for evaluating goals in mental health care. *AMA Journal of Ethics*, *25*(9), E690–E702. https://doi.org/10.1001/amajethics.2023.690

Westermair, A. L., Weber, S., Westmoreland, P., Mehler, P. S., Elsner, F., & Trachsel, M. (2024). Scoping review of end-of-life care for persons with anorexia nervosa. *Annals of Palliative Medicine*, *13*(3), 685–707. https://doi.org/10.21037/apm-23-522

Westmoreland, P., & Mehler, P. S. (2016). Caring for patients with severe and enduring eating disorders (SEED): Certification, harm reduction, palliative care, and the question of futility. *Journal of Psychiatric Practice*, *22*(4), 313–320. https://doi.org/10.1097/PRA.0000000000000160

Westmoreland, P., Parks, L., Lohse, K., & Mehler, P. (2021). Severe and enduring anorexia nervosa and futility: A time for every purpose?. *Psychiatric Clinics of North America*, *44*(4), 603–611. https://doi.org/10.1016/j.psc.2021.08.003

Xu, Y. E., & Sisti, D. (2021). Futility and terminal mental illness: The conceptual clarification continues. *Perspectives in Biology and Medicine*, *64*(1), 44–55. https://doi.org/10.1353/pbm.2021.0004

Yager, J. (2015). The futility of arguing about medical futility in anorexia nervosa: The question is how would you handle highly specific circumstances?. *American Journal of Bioethics,* *15*(7), 47–50. https://doi.org/10.1080/15265161.2015.1039724

Yager J. (2020). Managing patients with severe and enduring anorexia nervosa: When is enough, enough?. *Journal of Nervous and Mental Disease*, *208*(4), 277–282. <https://doi.org/10.1097/NMD.0000000000001124>

Zettl, R. E., & Sadler, J. Z. (2020). Psychiatric ethics. In E. Ryznar, A. B. Pederson, M. A. Reinecke, & J. G. Csernansky (Eds.), *Landmark Papers in Psychiatry* (pp. 329–345). Oxford, United Kingdom: Oxford University Press. [https://doi.org/10.1093/med/9780198836506.003.0020](https://psycnet.apa.org/doi/10.1093/med/9780198836506.003.0020).

Zhong, R., Xu, Y., Oquendo, M. A., & Sisti, D. A. (2019). Physician aid-in-dying for individuals with serious mental illness: Clarifying decision-making capacity and psychiatric futility. *American Journal of Bioethics*, *19*(10), 61–63. https://doi.org/10.1080/15265161.2019.1654018
